# Age-Markers on the Red Blood Cell Surface and Erythrocyte Microparticles may Constitute a Multi-parametric Strategy for Detection of Autologous Blood Transfusion

**DOI:** 10.1186/s40798-023-00662-9

**Published:** 2023-12-01

**Authors:** Giorgia M. Biasini, Francesco Botrè, Xavier de la Torre, Francesco Donati

**Affiliations:** 1 Sapienza University of Rome, Rome, Italy; 2 Laboratorio Antidoping, Federazione Medico Sportiva Italiana, Rome, Italy; 3 REDs – Research and Expertise in anti-Doping Sciences, ISSUL – Institute of Sport Sciences University of Lausanne, Lausanne, Switzerland

**Keywords:** Doping in sport, Blood doping, Autologous blood transfusions, Glycophorin-A, Band 3 complex, Peroxiredoxin-2, CD47, Phosphatidylserine, Red blood cell microparticles

## Abstract

**Background:**

Autologous blood transfusion is one of the illicit strategies, banned by the World Anti-Doping Agency, to increase the levels of hemoglobin, with a consequent improvement in the delivery of oxygen to tissues. At present, this practice is detectable exclusively by the individual, longitudinal monitoring of hematological biomarkers, as in the hematological module of the Athlete Biological Passport; but this indirect approach may suffer from different confounding factors. We are presenting a multi-parametric, analytical strategy to detect autologous blood transfusions by targeting the modification of the red blood cells during storage. We focused on the assessment of “storage lesions”, targeting (i) membrane proteins: Glycophorin-A and Band 3 complex, (ii) biomarkers of oxidative stress: Peroxiredoxin-2, (iii) biomarkers of senescence: CD47 and Phosphatidylserine, (iv) erythrocytes microparticles.

**Results:**

All of the above markers were monitored, by immunological and flow cytofluorimetric methods, on samples of stored whole blood collected at different time intervals, and on fresh blood samples, collected for official doping control tests, mixed “ex vivo” to simulate an autotransfusion. Although anonymized before the delivery to the laboratory, it was possible to mix samples belonging to the same subject based on the “athlete biological passport” code. Our results showed that the irreversible alteration of RBCs morphology, the loss of membrane integrity, the occurrence of hemolysis phenomena, and, more in general, the “aging” of the erythrocytes during storage are closely related to: (i) the reduced concentration, on the erythrocyte membrane, of Band 3 protein (decrease of 19% and of 39% after 20 and 40 days of storage respectively) and of glycophorin A (− 47% and − 63% respectively); (ii) the externalization of phosphatidyl serine (with a five-fold increase after 20 days and a further 2× increase after 40 days); (iii) the reduced concentration of CD47; and (iv) increased levels of erythrocyte microparticles.

**Conclusions:**

The most promising method to detect the presence of transfused blood in whole blood samples can be based on a multi-parametric strategy, considering jointly both protein expression on RBCs membranes and micro-vesiculation phenomena.

## Key Points

- Potential biomarkers of “blood storage” were specifically considered in this study to detect autologous blood transfusions in sport doping.
- The irreversible alteration of RBCs morphology, the loss of membrane integrity, the occurrence of hemolysis phenomena, and, more in general, the “aging” of the erythrocytes during storage are closely related to: (i) the reduction, on the erythrocyte membrane, of Band 3 protein and of glycophorin A; (ii) the externalization of phosphatidyl serine; (iii) the reduced concentration of CD47; and (iv) the increased levels of erythrocyte microparticles.
- Freshly collected samples showed differences from stored or mixed ones in terms of both protein expression on the RBCs membranes and micro-vesiculation phenomena.

## Background

The term “blood doping” indicates the misuse of substances or techniques aimed to increase the number of red blood cells (RBCs) in the bloodstream, improving oxygen delivery to muscles and enhancing athletic performance, especially in endurance sport disciplines. A wide group of banned practices are considered blood doping, including homologous (HBT) or autologous (ABT) blood transfusions of whole blood or erythrocytes concentrates (reviewed in [1]). Any manipulation of blood and/or blood components is considered doping and prohibited by the World Anti-Doping Agency (WADA) [2]. Officially approved and validated direct tests are available for the majority of the blood doping strategies, including HBT (reviewed in [3]), but one of the greatest challenges for anti-doping scientists is the development of a direct method to detect the recourse to ABT.

In details, ABT consist of the withdrawal of a certain amount of blood (typically, in the range 400–500 mL), its storage for a sufficient period (usually up to six weeks) to allow the full replacement of total blood volume, and its reinfusion a short time before the competition. Although the risks related to immunoreactions are extremely low in the case of ABT compared to HBT, the risk of acute cardiovascular complications is still relatively high. Moreover, inappropriate conditions of storage may lead to infections and other complications [4].

Currently, the only officially validated method for ABT detection is indirect: it is based on the individual, longitudinal monitoring of specific hematological parameters carried out in the framework of the hematological module of the Athletes’ Biological Passport (ABP) [5]. Values that are out of the athlete’s individual reference ranges can indicate the recourse to illicit substances and/or methods aimed to increase the transport of oxygen to the tissues. However, ABP has some limitations [6], such as the difficulty to detect micro-dosage transfusions of RBC [7], the recourse to masking agents (e.g. plasma volume expanders), and the generation of confounding profiles due to specific physical and/or environmental conditions (e.g. altitude and/or high-intensity training) [8, 9] (reviewed in [10]).

During storage, RBCs go through numerous irreversible changes, that involve membrane markers, cytoskeletal proteins, and enzymes, resulting in morphological, biochemical, and functional modifications, collectively defined as “RBC storage lesions” [11]. More specifically, erythrocytes undergo oxidative lesions, loss of ATP, changes in the metabolome and proteome [12] that cause modifications of shape and volume, and membrane integrity. It is widely known that during storage, the morphology of RBCs can be significantly modified: erythrocytes lose their characteristic discoidal shape and develop progressive spiculation on their surface (echinocytes), accruing spherical form (spherocytes) [13, 14]. The most probable sites of damage will be the cytoskeletal and membrane proteins [15]. Moreover, these membrane modifications lead to increased RBCs osmotic fragility and changes in electrolyte imbalance, resulting in augmented oxidative stress and damages of those enzymes responsible for the oxidant-antioxidant balance. Due to their altered shape and compromised functionality, RBCs experience early senescence and begin the apoptotic process. Furthermore, senescent erythrocytes undergo a reduction of their dimension, due to the phenomena of micro-vesiculation, with the parallel formation of RBCs microparticles (RMPs) [16, 17]. These damages involve structural, morphological, metabolic, and functional irreversible modifications.

In this study we focused on the RBCs “storage lesions” to develop a direct method for the detection of ABT, complementing the current indirect approach based on the hematological module of the Athlete Biological Passport, that, as anticipated above, is centered on the individual, longitudinal monitoring of some key hematological parameters. More specifically, we have considered four different aspects of storage damages, detailed below:

1. Changes regarding RBCs membrane proteins, such as Band 3 single protein and macro-complex and Glycophorin-A (GYPA), were investigated over storage time. Band 3 macro-complex represents the main erythrocytes transmembrane protein, and it was demonstrated to bind Rh complex [18] and various cytoskeletal proteins [19], having an important role in the maintaining of membrane integrity, cytoskeletal organization [15, 20] and in maintaining electrolyte imbalance [21]. The surface biomarker GYPA represents the major sialoglycoprotein of RBC membrane and it is generally considered to be specific to erythroid cells [22].
2. Alteration of Peroxiredoxin-2 (PRDX2), the third most abundant protein of RBCs, classified as biomarker of oxidative stress due to its antioxidant function. RBCs are constantly exposed to oxidative stress and PRDX2 function to reduce peroxides is very important for their survival [23]. During storage oxidative stress increases and PRDX2 function is impaired, due to the impossibility of the enzyme to return to its reduced form [24].
3. Biomarkers of RBCs senescence, such as Phosphatidylserine (PS) and CD47. PS is a phospholipid of RBC membrane. In the standard condition, it is in the inner leaflet of the plasmatic membrane, but it migrates to the outer side in case of senescent or damaged erythrocytes [25]. PS exposure on RBCs determines the beginning of the apoptotic phase and represents a clear signal for the macrophage for phagocytosis [26]. On the contrary, CD47 is a transmembrane protein involved in the regulation of cell survival [27]. A decline of cell surface CD47 expression during RBCs aging in vivo promotes the clearance of senescent RBCs [28]. During storage, in an *ex-vivo* context, the mechanism of clearance is impaired. For this reason, we selected PS and CD47 as biomarkers of RBCs senescence since they cannot be removed in simulated storage conditions.
4. Evaluation of the micro-vesiculation phenomena in RBCs over storage time. Among the irreversible “RBC storage lesions” there is a severe reduction of erythrocytes size, due to the formation of RMPs [29]. RMPs are membrane particles measuring around 1 μm, produced by stimulated or apoptotic erythrocytes through modulation of membrane lipid organization and cytoskeleton reorganization. RMPs express antigens characteristic of their original cell type [16]. In-vivo conditions, they are usually eliminated by the clearance mechanism, but during storage, this process is impaired, still being traceable after the reinfusion. In the present study, we investigated the variation of RBCs parameters described above, before, and after storage of blood samples under standard blood banking conditions.

Finally, we simulated ex-vivo blood transfusion experiments, assessing the relevance of the selected parameters as potential biomarkers of ABT.

## Methods

### Sample Collection and Storage

In compliance with the WADA International Standards for Laboratories [30], all samples selected for this study were from athletes who had priorly given consent to the use of their samples for research purposes, according to the information reported on the doping control form. In details, aliquots of samples from elite athletes, tested negative to antidoping screening tests, were collected in EDTA-treated tubes and enriched with 14% of citrate phosphate dextrose with adenine (CPDA, Sigma-Aldrich, USA) and stored under standard blood banking conditions at 2–8 °C for 20 (T1) or 40 days (T2). Samples were analyzed within 24-h from collection (T0) and/or after a fixed period of storage. To simulate an autologous blood transfusion, a group of samples was obtained by mixing freshly collected whole blood samples with 10% of stored blood from the same athlete (T2), following a procedure already described by our group [17]. The selection of blood samples from the same athlete was possible (still without knowing the identity of the athlete) thanks to the “biological passport code”: in other words, samples collected for the analysis of the hematological module of the athlete biological passport (ABP), although still anonymized, are coded in a way that it is possible to trace the samples collected from the same subject, without knowing his/her identity. To evaluate age-related differences, erythrocytes were separated by density, using Percoll (Sigma-Aldrich, USA) discontinuous gradients, according to the procedure described by Bosch et al. [31].

### Sample Analysis

All biomarkers considered in this study are listed in Table 1, that reports also the corresponding analytical techniques for their determination and the different storage times.

**Table 1:** Summary of the biomarkers, biological matrices and analytical techniques considered in the present study

Biomarkers	Matrix	Storage time (days)	Analytical technique
Glycophorin-A	Whole blood	0; 20; 40	Flow Cytofluorimetry
Band 3	Whole blood; erythrocytes fractions	0; 20; 40	Flow Cytofluorimetry; Western Blot
Peroxiredoxin-2	Whole blood; erythrocytes fractions	0; 20; 40	Western Blot
CD47	Whole Blood	0; 20; 40	Flow Cytofluorimetry
Phosphatidylserine	Serum; mixed	0; 20; 40; Mixed (90% t0 + 10% t40)	ELISA assay
Red Blood Cells Microparticles	Whole blood; mixed	0; 20; 40; Mixed (90% t0 + 10% t40)	Flow Cytofluorimetry

#### Flow Cytofluorimetry Analysis

Whole blood samples were diluted in PBS to reach the concentration of 1 million RBCs/100 μL. Aliquots of 100 μL were stained with 5 μL of specific primary antibodies: anti-Glycophorin-A-PE (Beckman Coulter Srl, Italy), anti-Band3-FITC (ThermoFisher, USA), anti-CD47-PE (ThermoFisher, USA) or eosin-5’-maleimide dye (1:5 E5M, ThermoFisher, USA). Samples were incubated for 45 min at RT, washed, and assayed by the flow cytofluorimeter (Cytomics FC500, Beckman Coulter, USA). The settings used were the following: Forward Scatter (FS) discriminator set at the detector channel 20; 50000 events/sample.

**Fig. 1:**
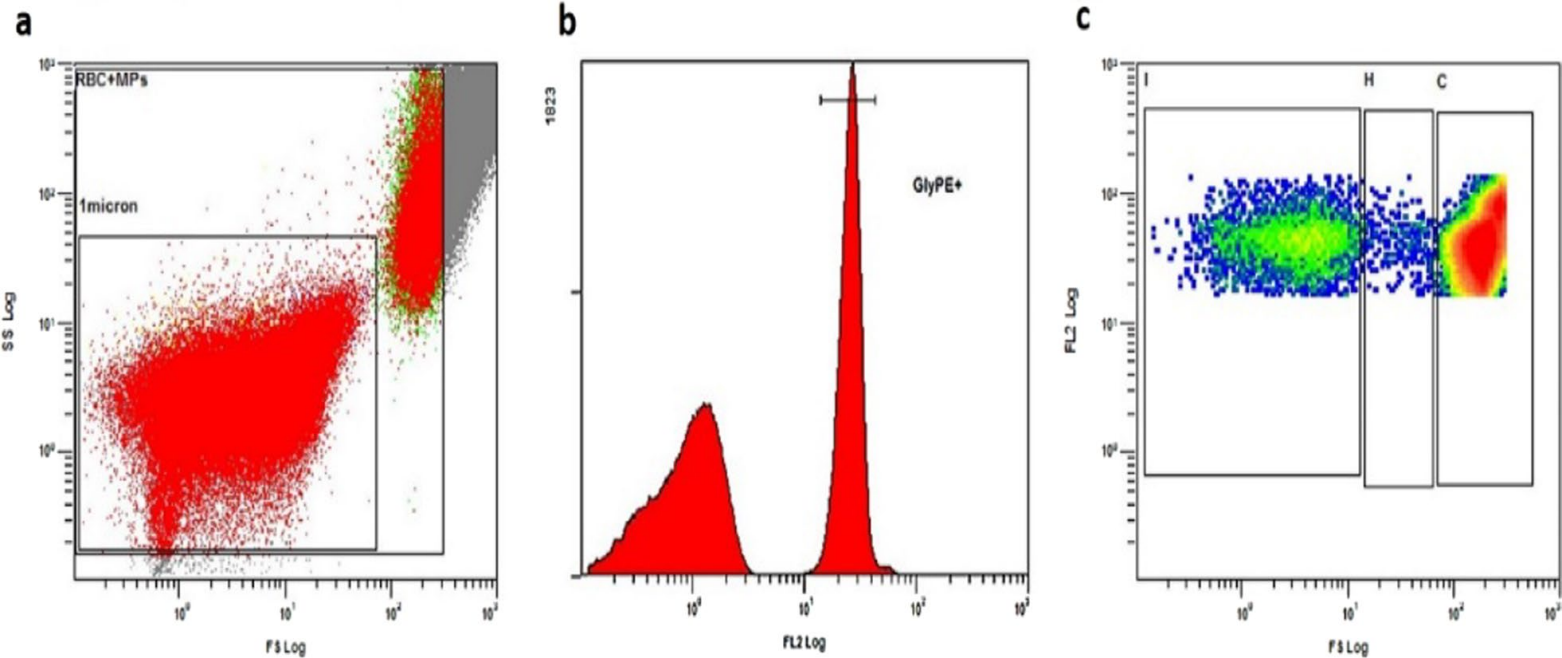
Flow cytofluorimetry gate settings for red blood cells (RBC) microparticles (RMPs) analysis. **a** Setting of a gate based on cell dimension (SS) and complexity (FS), which allows to include into the analysis only cell ≤ 1 μm. **b** Setting of a gate based on FL2 detector and cell binding to Glycophorin-A (GYPA)-PE antibody. **c** Setting of gates based on GYPA expression and dimension. Gate C defines RBCs, gate H identifies the Transitional Events (TE), while gate I identifies events expressing GYPA smaller than 1 nm, representing RMPs

To analyze RMPs, it was developed a time-saving approach that allows RMPs identification without extraction [29, 32]. Proper gates were set on CXP Analysis Software (Beckman Coulter, USA), according to cell morphology and surface protein expression. Fluorescent microspheres (Beckman Coulter, USA) were used to include in the analysis only events of 1μm or lower, which is the conventional diameter of RMPs [33] (Fig. 1a). The surface expression GYPA was also considered (Fig. 1b) and three gates were defined to identify RBCs (Fig. 1c, gate C), transition events (TE) (Fig. 1c, gate H), and RMPs (Fig. 1c, gate I).

#### Western Immunoblotting Assay

Ghost membrane extraction was performed according to Low et al. [34]. Proteins, separated by SDS-PAGE, were transferred to a PVDF membrane (Immobilon, Italy) and stained with primary antibodies against PRDX2 (1:1600, R&D Systems, USA) and Band 3 protein (1:100,000, ThermoFisher, USA). Membranes were then incubated with secondary antibody (Gt anti-MS IgG, HRP-conjugate, ThermoFisher, USA). After the substrate addition, images were acquired with ImageQuant LAS400 (GE-Healthcare, USA). Bands of interest were quantified by densitometry utilizing the GasEPO software (WADA).

#### ELISA Assay

PS levels were measured through an enzyme-linked immunosorbent assay (ELISA) sandwich kit (LifeSpan Biosciences, USA). Plates were read on a Victor^3^ Multilabel Plate Reader (Perkin Elmer, USA) at 450 nm.

### Data Analysis

Basic statistics was performed using Statistica 12.0 (Statsoft, USA) and GraphPad Prism (GraphPad Software Inc, USA) softwares. To compare the mean values of the RBCs parameters within different experimental group, repeated measures analysis of variance (ANOVA) was used. Tukey HSD test was used as a post-hoc analysis. Statistical significance was set at *p* < 0.05.

## Results

### RBCs Surface Markers and Membrane Proteins

#### Band-3 Protein and Band-3 Complex

**Fig. 2:**
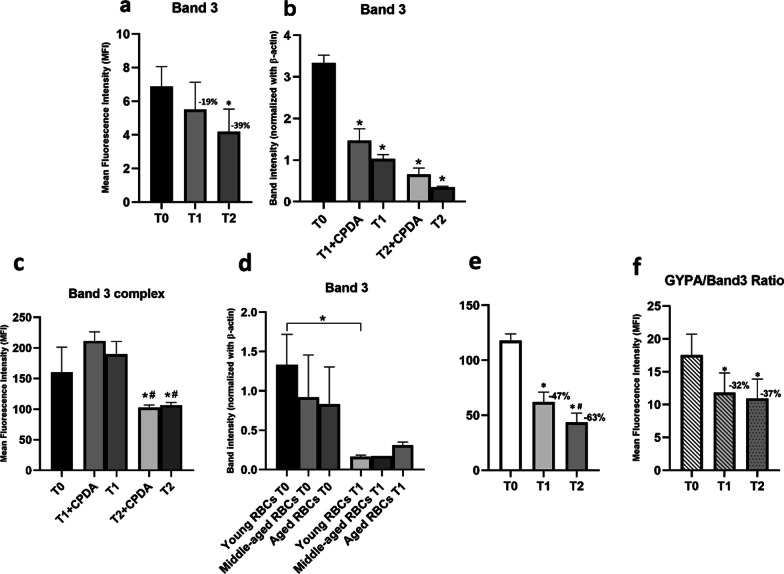
Changes of Band 3 (protein and/or complex) and of GYPA concentrations as a function of storage time. **a** Band 3 levels measured by flow cytofluorimetry; **b** Band 3 levels measured by western immunoblotting assay; **c** Band 3 complex levels measured by flow cytofluorimetry analysis, using Eosin-5’-maleimide dye, which binds the whole macro-complex and not only the single protein. **d** Band 3 protein levels measured by western immunoblotting assay in RBCs fractions isolated by Percoll density gradients; **e** GYPA levels measured by flow cytofluorimetry **f** GYPA/Band 3, both measured by flow cytofluorimetry

The trend of the levels of Band-3 protein during storage time is reported in Fig. 2a (flow cytofluorimetric assays) and in Fig. 2b (western immunoblotting assay), while Fig. 2c reports the trend of the levels of the Band 3 complex: in this case, we did not use a specific antibody for the Band 3 protein, but the E5M dye, which binds to a *ξ*-NH2 group of Lys-430 on Band 3 macrocomplex and a deficiency in Band 3 results in a decrease of fluorescence intensity [35]. Finally, Fig. 2d shows the levels of Band-3 protein over storage time from the western immunoblotting assay in RBCs fractions isolated by Percoll density gradients.

#### GYPA

GYPA levels were analyzed by flow cytofluorimetry in freshly collected and stored blood samples. The results are shown in Fig. 2e.

#### GYPA/Band 3 Ratio

The trend over time of the ratio between GYPA levels and Band 3 protein, measured as described above, is shown in Fig. 2f).

### Biomarkers of Oxidative Stress: PRDX2

**Fig. 3:**
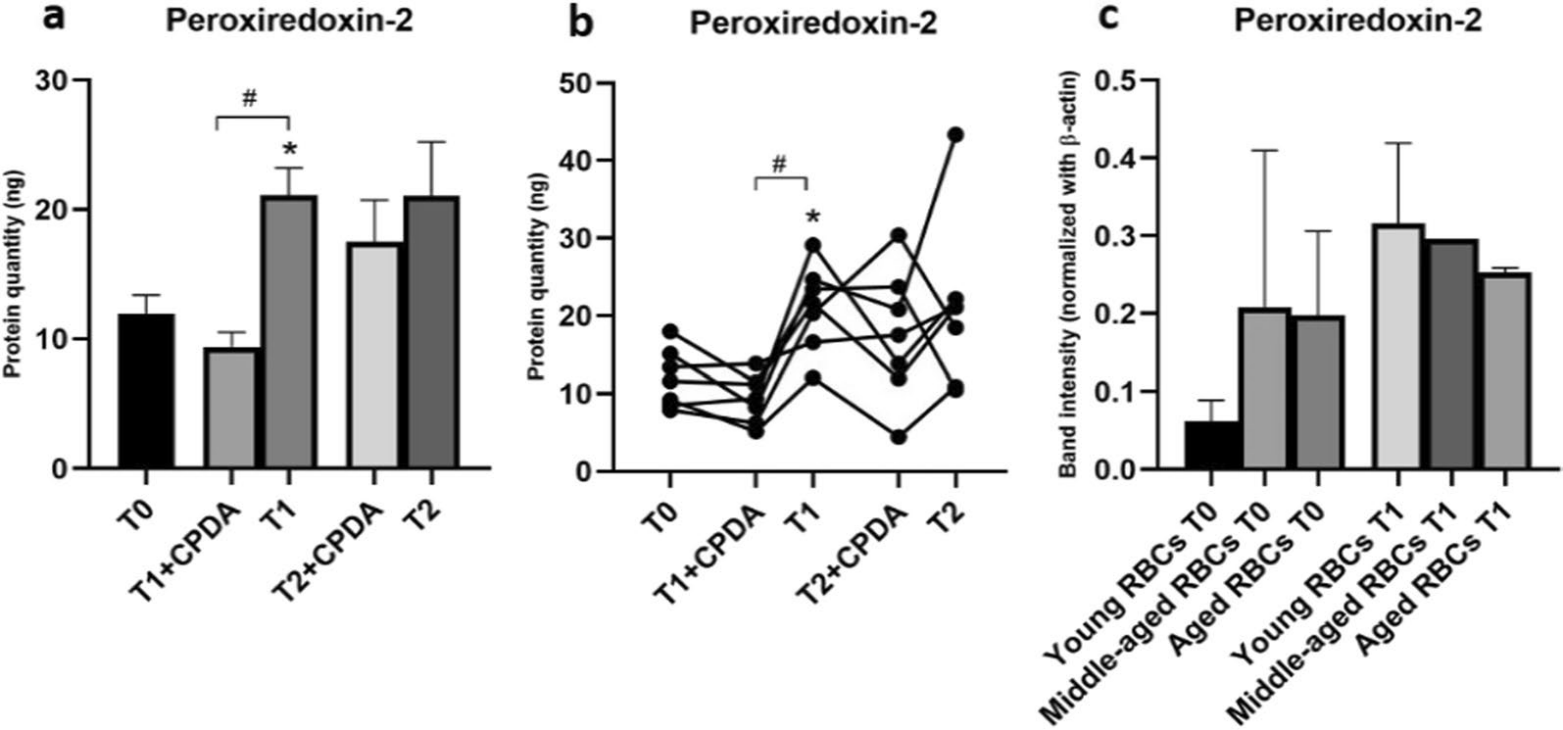
Change of expression of PRDX2 as a function of storage time, assessed by western immunoblotting assay of the ghost membrane of whole blood samples and RBCs fractions. **a** PRDX2 levels during storage both in the presence and in the absence of CPDA; **b** PRDX2 levels in RBCs fraction isolated by density

Since PRDX2 is located in the cytosol, to allow the PRDX2 specific antibody to cross the membrane and bind to the target protein, we initially explored the possibility of performing a preliminary fixation and permeabilization pretreatment of the RBC before the flow cytofluorimetric assay [36]. Unfortunately, due to the increased fragility of RBCs over storage time, this process did not allow to obtain repeatable results, and the differential sensitivity was overall unsatisfying. The levels of PRDX2 in the RBCs before, during, and after storage were therefore assessed on erythrocytes ghost membranes by western immunoblotting assay, which is widely used for its analysis [37–39]. Figure 3a and b show respectively the progressive increase of PRDX2 concentration of individual samples over storage time.

### RBCs Markers of Senescence

#### CD47

**Fig. 4:**
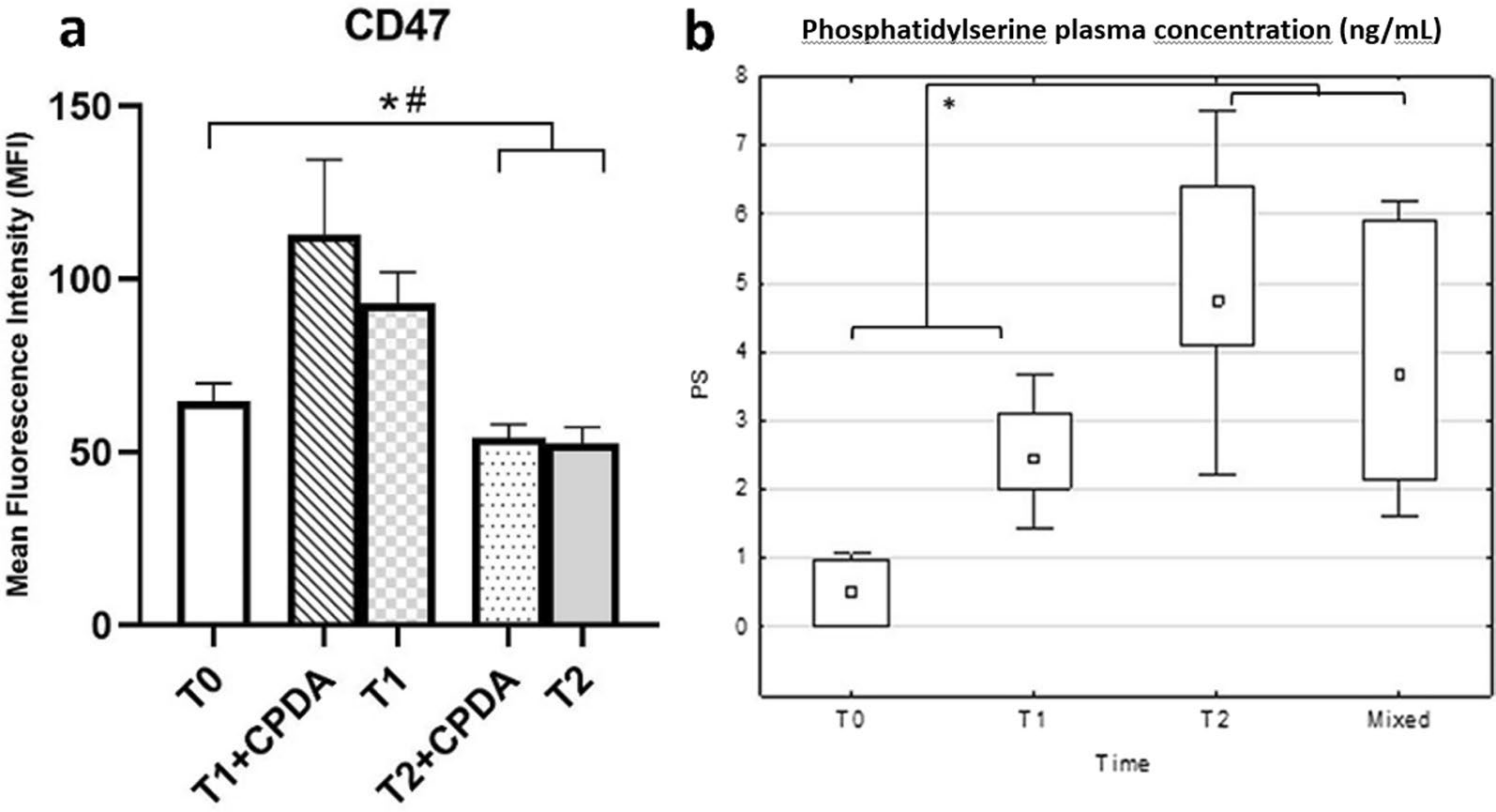
Change of expression of CD47 and Phosphatidylserine (PS) over storage time. **a** CD47 levels measured at T0, T1 and T2 in the presence and in the absence of CPDA; **b** PS concentration in freshly collected blood samples (T0), stored samples, respectively for 20 days (T1) and 40 days (T2), and mixed samples (10% sample stored for 40 days + 90% fresh sample)

Figure 4a shows the trend of CD47 concentration in whole blood samples (n = 9) as a function of the storage time.

#### Phosphatidylserine

PS levels were evaluated in freshly collected (n = 9), stored (T1 n = 13, T2 n = 13) and in mixed samples (n = 8) by a sandwich ELISA assay. The results are shown in in Table 2 and in Fig. 4b.

**Table 2:** Mean values of phosphatidylserine (PS) concentration in the different experimental groups, with related standard error and the sum of the mean ± 95% of the standard error

Time	PS mean	PS std error	PS − 95%	PS + 95%	N
T0	0.48	0.43	− 0.39	1.36	9
T1	2.51	0.36	1.78	3.23	13
T2	5.01	0.36	4.28	5.74	13
Mixed	3.91	0.46	2.98	4.84	8

### RMPs Formation

**Fig. 5:**
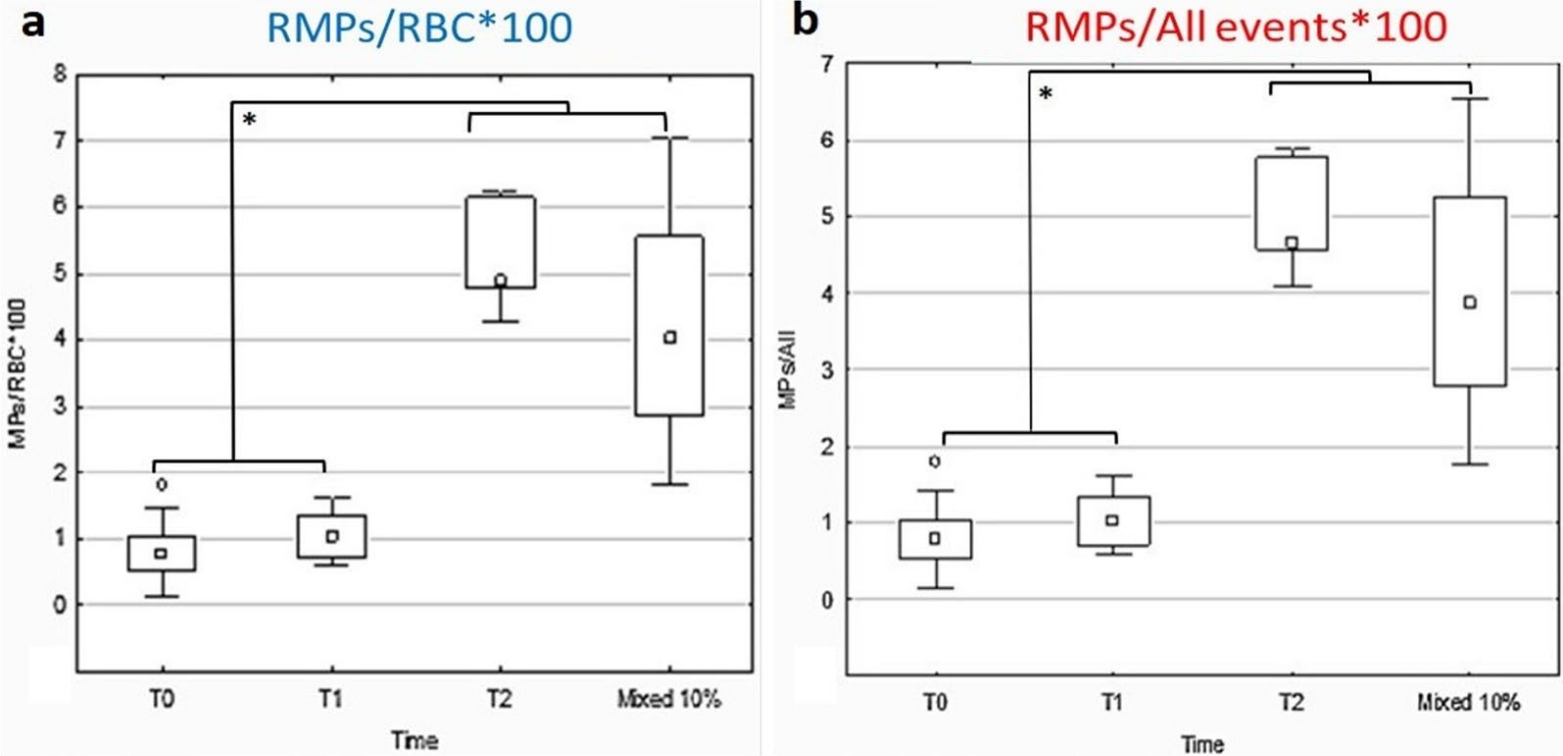
Red blood cells (RBC) microparticles (RMPs) formation over storage time from flow cytofluorimetry assay in whole blood samples freshly collected, stored with citrate phosphate dextrose with adenine (CPDA) solution or transfused ones. **a** RMPs/RBCs*100 ratio increases after 40 days of storage (T2) (*, *p* < 0.001) and in Mixed 10% samples (*, *p* < 0.001) compared to freshly collected samples. No differences were found after 20 days of storage (T1). **b** RMPs/All events*100 ratio increases after 40 days of storage (T2) (*, *p* < 0.001) and in artificially transfused samples (Mixed 10%) (*, *p* < 0.001) compared to freshly collected samples. No differences were found after 20 days of storage (T1)

RMPs formation in freshly collected (n = 30), stored for 20 (T1, N = 16) and 40 days (T2, n = 16), and “Mixed 10%” (n = 24) whole blood samples was analyzed without extraction, with a gating setting through FC. The results, expressed by the mean of the morphological events counted within the gate (< 1 µm), are reported in Table 3. Figure 5 shows the RMPs events expressed as a percentage of RMPs/RBCs (a) and RMPs/All events (b) ratios in freshly collected, stored with CPDA solution and mixed samples.

**Table 3:** Mean values of the concentration percentage of RMPs/RBC and RMPs/All events in the different experimental groups, with related standard error

Time	RMPs/RBC*100 mean	RMPs/RBC*100 std error	RMPs/All*100 mean	RMPs/All*100 std error	N
T0	0.78	0.19	0.77	0.17	30
T1	1.05	0.37	1.04	0.34	8
T2	5.16	0.40	4.90	0.36	7
Mixed	4.23	0.21	4.02	0.19	24

**Fig. 6:**
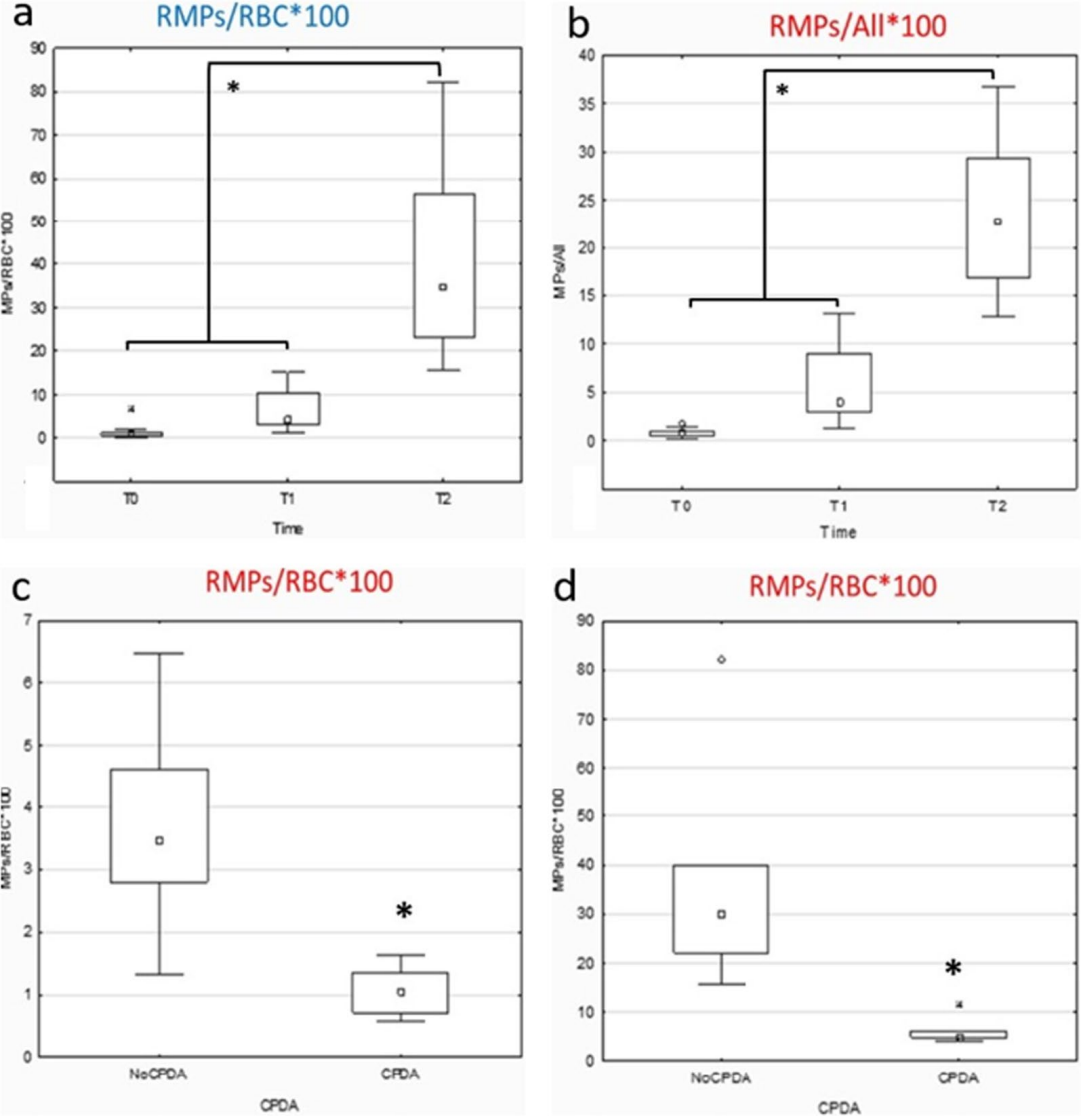
Differences in Red blood cells (RBC) microparticles (RMPs) formation during storage, bith in the presence and in the absence of CPDA. **a** RMPs/RBCs*100 ratio; **b** RMPs/All events*100 ratio **c** RMPs/RBCs* ratio after 20 days of storage with and without CPDA; **d** RMPs/All events*100 ratio with and without CPDA

In addition to the above, we also measured the levels of RMPs in freshly collected blood samples and in stored samples without CPDA solution (Figs. 6a and 6b). Figure 6 c and d show the box plot of the percentage of the mean value of RMPs/RBCs ratio, respectively after 20 days (Fig. 6c) and 40 days of storage (Fig. 6d), with and without CPDA.

**Fig. 7:**
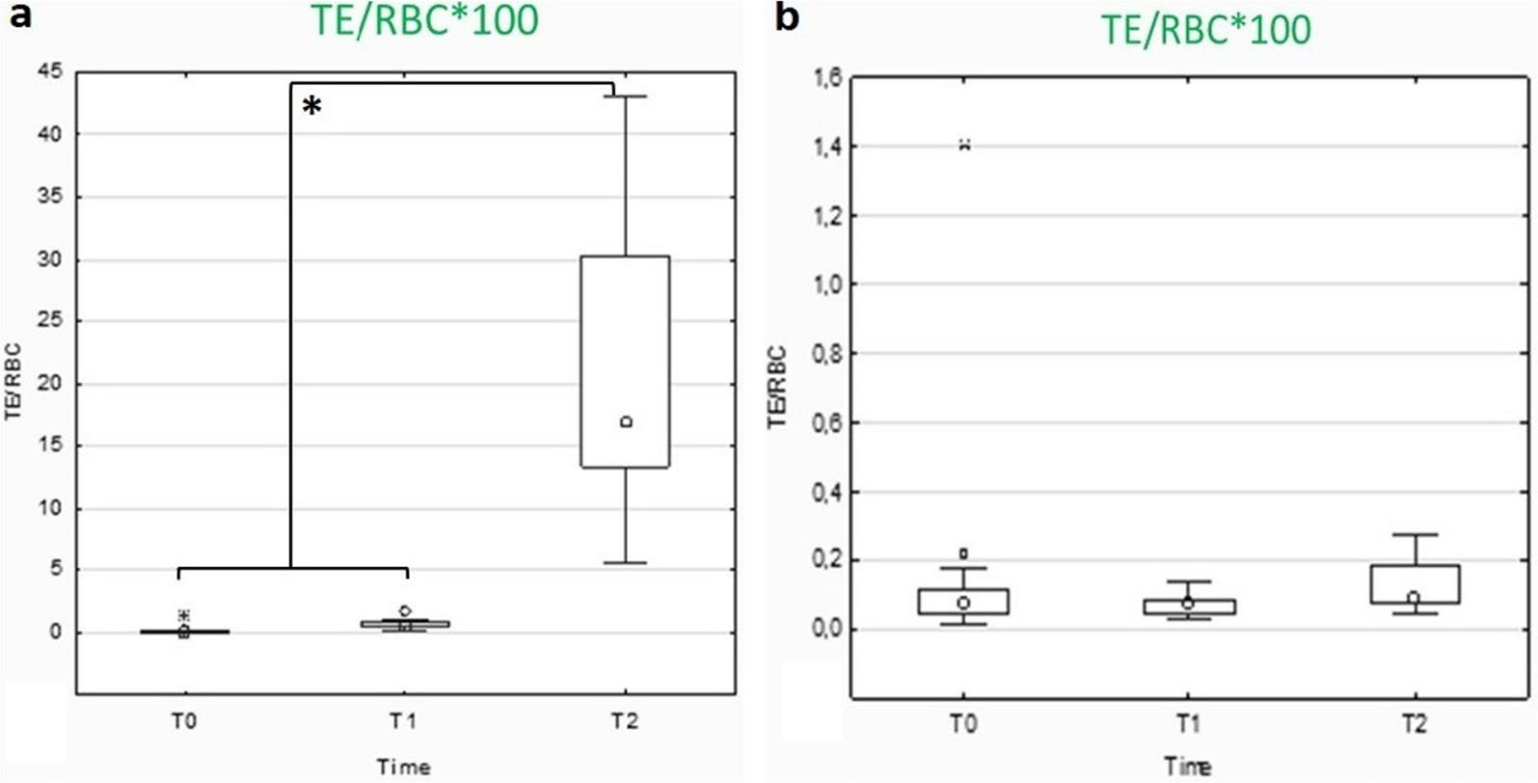
Changes in transitional events (TE) concentration over storage time in whole blood samples stored with (**a**) or without (**b**) CPDA

Finally, the effect of preservatives (in our case, CPDA) on the formation of transition events (TE), was also assessed (Fig. 7).

## Discussion

The development of innovative methods to detect the recourse to HBT and ABT has always represented a challenge in antidoping analysis. To complement the effectiveness of the indirect testing procedures based on the hematological module of the ABP, we have designed a multi-parametric approach, based on the changes that occur in the RBC during storage. “Storage lesions” originate from biochemical events that lead to irreversible changes and alterations in long-term stored RBCs after blood banking. In the past, we have addressed the issue of micro-vesiculation, with release of RBC microparticles, as a marker of blood storage to be considered in doping control analysis [17, 29]. We have here considered, more specifically also some among the main biochemical changes, occurring mainly on the RBC membrane, when whole blood is stored at 4 °C for a sufficiently long time. Specifically, alterations during storage were reported for Band 3 protein [20, 40–42], GYPA [42, 43], PRDX2 [24, 37, 38, 43–45], CD47 [46, 47] and phosphatidylserine [25, 26, 48]. The biomarkers we have considered and measured in freshly collected, stored, and mixed samples are discussed here below.

### RBCs Surface Markers and Membrane Proteins

#### Band-3

Band 3 protein is a component of the Band 3 macro-complex, which binds various other membrane and cytoskeletal proteins. The levels of Band-3 protein, assayed by flow cytofluorimetry, show a decrease by 19% after 20 days (T1), and of 39% after 40 days (T2) of storage (*, *p* = 0.01) (Fig. 2a). These data were confirmed by western immunoblotting assay, performed on extracted erythrocytes ghost membranes [30]: Band-3 levels were found to decrease at T1 both with (*, *p* = 0.01) and without (*, *p* < 0.0001) citrate phosphate dextrose with adenine (CPDA) solution (Fig. 2b). The decrease is more evident at T2, both with (*, *p* < 0.0001) and without (*, *p* < 0.0001) CPDA. C).

As shown in Fig. 2c, the presence of CPDA protects the integrity of the RBCs over 20 days of storage, but it is not sufficient to prevent RBCs membrane damages after 40 days. Band-3 complex levels decrease with statistical significance after 40 days both with and without the addition of CPDA solution. Interestingly, while Band 3 single protein levels have shown a trend of decrease after 20 days of storage without CPDA solution (see again Fig. 2a), this phenomenon is reverted when the whole Band 3 macro-complex is analyzed.

Finally, the concentration of Band-3 protein was estimated in young, old, and middle-aged erythrocytes, to assess whether the changes recorded during storage were correlated with an accumulation of senescent RBCs, caused by impaired clearance in the *ex-vivo* conditions. Results are shown in Fig. 2d. As it can be seen, there is a trend of decreasing in Band-3 protein levels comparing old RBCs to younger ones, consistently with our hypothesis of the reproducibility of Band 3 expression pattern in stored samples and aged erythrocytes. Moreover, our results show that Band-3 levels in young erythrocytes after 20 days of storage are lower than those measured in freshly collected samples, with statistical significance (*p* < 0.05). Unfortunately, results after 40 days could not be obtained due to the fragility of the cells, showing severe age-related degradation phenomena.

#### GYPA

Glycophorin-A (GYPA) levels were analyzed by flow cytofluorimetry. The results are expressed as the average value of the Mean Fluorescence Intensity (MFI) of seven different blood samples. GYPA levels were found to decrease after 20 days (T1) (*, *p* < 0.0001) and after 40 days (T2) (*, *p* < 0.0001) of storage compared to freshly collected samples (T0). GYPA resulted in a progressive decrease during storage (47% after 20 days and 63% after 40 days). GYPA levels decreased significantly also between 20 and 40 days of storage (Fig. 2e).

#### GYPA/Band 3 Ratio

The ratio GYPA/Band 3, both measured by flow cytofluorimetry, changes during storage, showing a reduction by 32% and 37%, respectively, after 20 and 40 days of storage, with statistical significance both between T0 and T1 and T0 and T2 (Fig. 2f).

### Biomarkers of Oxidative Stress: PRDX2

PRDX2 showed an inverted pattern of expression over storage time: for indeed, PRDX2 levels were shown to increase after 20 days of storage and this phenomenon is even more evident without CPDA solution. Results shown in Fig. 3a indicate that there is an augmentation in PRDX2 levels immediately after 20 days, with statistical significance (*p* < 0.05). This increase is maintained after 40 days, but without a progressive trend of growth. These results can be explained by an accumulation of PRDX2 due to the increased storage-dependent oxidative stress. In these conditions the reduction of oxidized PRDX2 is impaired, resulting in the loss of the protein antioxidant function. PRDX2, then, accumulates in its oxidized form, migrating from the cytosol to the membrane, becoming more detectable by the analysis of RBCs ghost membranes through western blot.

PRDX2 levels were also analyzed in RBCs fraction isolated by density, to assess whether PRDX2 concentration was higher in older RBCs as well as in stored ones. No differences were found between aged and young RBCs membranes (Fig. 3c).

### RBCs Markers of Senescence

#### CD47

Figure 4a shows the trend of CD47 concentration in whole blood samples as a function of the storage time. As it can be seen, levels of CD47 initially increase, to progressively decrease after prolonged storage. Levels at T1 were significantly higher than those at T0, regardless the presence of CPDA; while levels at T2 are lower than those at T0, again with no differences due to the presence of CPDA.

#### Phosphatidylserine

PS levels were measured, by a sandwich ELISA assay, in freshly collected, stored, and mixed samples. Our data indicate the progressive formation of apoptotic cells over storage time. Figure 4b shows that the expression of PS is higher in stored samples, already after 20 days of storage. PS levels become even higher after 40 days of storage and this increase is still traceable in mixed samples (“Mixed 10%”, prepared mixing 90% fresh blood and 10% blood stored for 40 days). The mean population values of PS concentration were calculated and reported in Table 2, with the related standard error and the sum of the mean ± 95% of the standard error. For both experiments, Tukey HSD post-hoc analysis highlighted a statistically significant difference between PS concentration between T0 samples and T1, T2, and mixed samples.

### RMPs Formation

RMPs formation in freshly collected, stored, and “Mixed 10%” whole blood samples was analyzed without extraction, with a gating setting through FC. The results are expressed by the mean of the morphological events counted within the gate (< 1 µm). Figure 5 shows the RMPs events expressed as a percentage of RMPs/RBCs (a) and RMPs/All events (b) ratios in freshly collected, stored with CPDA solution and mixed samples. Our results show the increase in RMPs concentration in stored (T2) or mixed samples. These data suggest that RMPs might be still traceable in transfused samples, although their concentration is higher in T2 samples. On the contrary, no differences in RMPs concentrations are detectable between freshly collected samples and samples analyzed after 20 days of storage with the addition of CPDA, resulting in a sudden increase in RBCs micro-vesiculation phenomena between 20 and 40 days.

Further information can be obtained also by following the transition events (TE). TE/RBCs and TE/All events ratios remain stable over storage time. Flow cytofluorimeter settings allowed to count a maximum of 100,000 events in each gate and the total amount of events (“All” events). RMPs/RBCs, RMPs/All events, TE/RBCs, TE/All events, and relative percentages were analyzed. The mean values of the population regarding percentages RMPs/RBCs and RMPs/All events ratios were calculated and reported in Table 3, with the related standard error. For both experiments, Tukey HSD post-hoc analysis highlighted a statistically significant difference between RMPs concentration between T0 samples and both T2 samples stored with CPDA solution and Mixed 10%, while no differences were found between T0 and T1 samples.

In addition to the above, we also measured the levels of RMPs in freshly collected blood samples and in stored samples without CPDA solution. As expected, RMPs formation over storage time is higher without the addition of CPDA solution and it is detectable even after 20 days, but with clearer and statistically significant results after 40 days (Fig. 6a and b).

Figure 6c and d show the box plot of the percentage of the mean value of RMPs/RBCs ratio, respectively after 20 days (Fig. 6c) and 40 days of storage (Fig. 6d), with and without CPDA. RMPs formation is significatively higher without the addition of CPDA solution, with higher values after 40 days, demonstrating a reduction of the percentage of RBC showing “storage lesions” in presence of a blood preservative.

Finally, the role of preservatives (in our case, CPDA), was also assessed: while no differences in TE/RBCs ratio percentage were highlighted between groups when samples were stored with CPDA solution, it was demonstrated that this ratio increases significantly (*p* < 0.05) when samples were stored without CPDA solution (Fig. 7).

The data we obtained allow the following considerations:

(i) The irreversible alteration of RBCs morphology, the loss of membrane integrity, the occurrence of hemolysis phenomena, are closely related to the reduced concentration of Band 3 protein and GYPA in the erythrocyte membrane over storage time, highlighting the suitability of those two proteins as biomarker of “RBC storage lesions”.
(ii) Erythrocytes undergo a fastened senescence and apoptotic process due to progressive, storage-dependent oxidative stress, revealed by the increase of PS-exposing RBCs and the reduced concentration of CD47. The combined analysis of those two biomarkers may help anti-doping authorities to detect the presence of transfused blood in whole blood samples.
(iii) A newly developed method, based on a system of gating by flow cytofluorimetry, allows to follow the formation of RMPs. This approach resulted to be efficient and timesaving, avoiding the complex procedure of RMPs extraction and increasing the repeatability of the method. The efficacy of this approach allowed us to define a threshold of RMPs concentration to identify transfused samples, with an accuracy of 75%. The actual applicability of this approach should be implemented with further experiments, but it may already be used by the anti-doping laboratories as a fast-screening strategy, activating the more complex procedure for the extraction of RMPs only in case of suspicious results.
(iv) Based on the above, the most promising method to discriminate freshly collected samples from stored or transfused ones should be based on a multi-parametric strategy, considering jointly both protein expression on RBCs membranes and micro-vesiculation phenomena.

Although of utility for the development of a multiparametric strategy for the detection of autologous blood transfusions, the present pilot study has two major limitations, outlined below:

(i) The biomarkers of storage investigated in this study were analyzed in freshly collected, stored, and “artificially transfused” blood samples (obtained, as previously described, through an *ex-vivo* mixture constituted by 90% freshly collected blood sample and 10% stored blood sample). Our results need therefore to be confirmed also on samples collected after a real transfusion, to assess the suitability of the selected biomarkers for the development of a novel method to detect the illicit recourse to ABT.
(ii) The pool of the tested samples in this study was limited to those samples belonging to athletes that resulted negative in the context of the anti-doping analysis and who gave their preliminary consent for their samples to be used for research purposes. The significance of our observation needs to be confirmed on a larger population of subjects/samples.

## Conclusions

To the best of our knowledge, this is the first study considering the modification of red blood cells during ex-vivo storage from the point of view of doping control analysis. A series of biomarkers of blood storage were considered to detect, by a multi-targeted approach, the modifications occurring on the RBCs as a function of the storage time. The results obtained in this study, although preliminary, offer a broad framework of data allowing to hypothesize the possibility of detecting those changes after their reinfusion. Increasing the pool of the tested samples will allow to further verify the solidity and real applicability of such a multi-targeted approach, also allowing to highlight more pronounced and statistically significant differences between freshly collected and transfused samples.

## Data Availability

Data sharing is not applicable to this article as no datasets were generated or analyzed during the current study.

## Abbreviations

ABT: Autologous blood transfusion
CD47: Cluster of differentiation 47
CPDA: Citrate–phosphate–dextrose anticoagulant solution
FC: Flow cytofluorimetry
GYPA: Glycophorin A
HBT: Homologous blood transfusion
IAP: Integrin Associated Protein
PRDX2: Peroxiredoxin-2
PS: Phosphatidylserine
RBC: Red blood cell
RMP: Red blood cell microparticle
TE: Transition event
